# The effects of heading time on yield performance and *HvGAMYB* expression in spring barley subjected to drought

**DOI:** 10.1007/s13353-023-00755-x

**Published:** 2023-03-10

**Authors:** Piotr Ogrodowicz, Anetta Kuczyńska, Paweł Krajewski, Michał Kempa

**Affiliations:** Institute of Plant Genetics, Polish Academy of Sciences, 34 Strzeszynska street, 60-479 Poznan, Poland

**Keywords:** Drought, Gibberellins, HvGAMYB, Barley, Earliness

## Abstract

In the lifetime of a plant, flowering is not only an essential part of the reproductive process but also a critical developmental stage that can be vulnerable to environmental stresses. To ensure survival during drought, plants accelerate the flowering process, and this response is known as “drought escape.” *HvGAMYB*—transcription factor associated, among others, with flowering process and anther development in barley—has also an important role in developmental modification and yield performance in plants subjected to stressed conditions. Due to the fact that information about the mechanisms associated both with the flowering acceleration and the anther or pollen disruption is limited, the exploration of the potential *HvGAMYB* role in flower development may shed light on pollen and spike morphology formations in plants grown under unfavorable water conditions. The aim of this study was to characterize differences in responses to drought among early- and late-heading barley genotypes. These two subgroups of plants—differentiated in terms of phenology—were analyzed, and traits linked to plant phenotype, physiology, and yield were investigated. In our study, the drought stress reactions of two barley subgroups showed a wide range of diversity in terms of yield performance, anther morphology, chlorophyll fluorescence kinetics, and pollen viability. The studied plants exhibited different yield performances under control and drought conditions. Moreover, the random distribution of genotypes on the biplot showing variability of OJIP parameters in the second developmental point of our investigation revealed that prolonged drought stress caused that among early- and late-heading plants, the studied genotypes exhibited different responses to applied stress conditions. The results of this study also showed that the *HvGAMYB* expression level was correlated positively with traits associated with lateral spike morphology in the second developmental point of this investigation, which showed that this association occurred only under prolonged drought and highlighted the drought stress duration effect on the *HvGAMYB* expression level.

**Supplementary Information:**

The online version contains supplementary material available at 10.1007/s13353-023-00755-x.

## Introduction

Climate data show that the frequency of droughts has increased over the past century, and an increase in the impact of droughts on crop productivity has been predicted (IPCC 2021). Therefore, understanding the effects of droughts on crop development and productivity is important.

During the stem elongation phase, appropriate irrigation conditions are essential for the development of fertile flowers at anthesis (Miralles and Slafer 1995). Water deprivation during this critical phase of development affects various aspects of plant metabolism and results in the impairment of many biochemical processes (Loggini et al. 1999; Farooq et al. 2009). The simplest way to withstand arid environmental conditions is to escape from drought (drought escape—DE) (Passioura, 1996).

Physiological tolerance to drought requires that the plant maintains vigor to produce a minimum number of seeds or simply survive, while agronomic tolerance requires maintaining an economically acceptable yield (Schafleitner et al. 2007). In the life cycle of plants, pollen development is an important stage because only normally formed pollen contributes to proper fertilization and formation of seeds and fruits. The signaling pathways that promote the transition to flowering include the following: the photoperiod pathway, hormone biosynthesis and signaling (GA), the pathway that acts independently of light (autonomous pathway), the pathway associated with low temperatures (vernalization pathway), the thermosensory pathway, and the age pathway (Halliday et al. 2003; Moon et al. 2005; Wang 2014; Ma et al. 2021). Previous studies have mapped the quantitative trait loci (QTL) associated with the heading date in all barley chromosomes (Pillen et al. 2003; Mikołajczak et al. 2016; Ogrodowicz et al. 2017).

The previous investigations (Gocal et al. 2001; Tsuji et al. 2006) suggest that *GAMYB* is a part of the central GA pathway, not only in the cereal aleurone layer but also in other plant tissues. This transcription factor (TF) encodes gibberellin-induced regulatory protein that is engaged in plant development and involved, among others, in anther formation (Bienias et al. 2020). In these organs, *HvGAMYB* levels are approximately doubled in response to GA3 (Aya et al. 2009). Moreover, *HvGAMYB* overexpression results in reduced anther length, lack of anther dehiscence, and male sterility, mimicking the effect of excessive GA on flower development (Zhang et al. 2020). A series of studies have reported the differing roles of *GAMYB* in the regulation of flowering (Yang et al. 2012; Zhang et al. 2020; Yang et al. 2021). For example, *GAMYB* expression accelerates flowering in gloxinia (*Sinningia speciosa*) (Li et al. 2013), but delays flowering in tobacco (*Nicotiana tabacum*) (Gallego-Giraldo et al. 2007). *GAMYB* encodes an R2R3-MYB transcription factor, which belongs to MYB transcription factor family playing vital roles in plant growth and development, including defense, cell differentiation, secondary metabolism, and responses to biotic and abiotic stresses (Tsuji et al. 2006; Zhang et al. 2020). For example, *AtMYB33*, *AtMYB65*, and *AtMYB101* in *Arabidopsis thaliana* play important roles in flower development, especially in pollen development (Gocal et al. 2001).

The aims of this study were to (i) investigate the effects of drought on phenologically differentiated spring barley plants and (ii) explore the expression pattern of the studied transcription factor (*HvGAMYB*) in anthers of early- and late-heading plants grown under drought conditions.

## Materials and methods

### Plant material, growth conditions, and experimental setup

In this study, six recombinant inbred lines (RILs) (hereafter referred to as LCam lines—LCam08, LCam12, LCam13, LCam37, LCam64, LCam71) and their parents (Lubuski and Cam/B1/CI08887//CI05761—hereafter referred as CamB) were examined. The plant material was developed following Mikołajczak et al. (2016). Quench was used as the reference variety.

All experiments were performed in growth chambers under fully controlled conditions (IPG PAS phytotrons). Five seeds from each of the accessions were sown in plastic pots (40 cm × 26 cm × 26 cm) filled with arable soil and peat (3:1, w/w), and the plants were cultivated under optimal conditions: temperature of 22 °C/18 °C day/night, humidity of 50–60%, and photoperiod of 16/8 h light/dark. Each treatment was conducted in triplicate. Barley external developmental stages’ scale was used (Gomez and Wilson 2012), in which the later stages of the Zadoks scale (Zadoks et al. 1974) were replaced by the last flag extension (LFE) stages to achieve a good system for reproductive development observation. The samples were collected at the following stages: LFE1 (flag leaf fully emerged and uncoiling—development point 1) and LFE3 (flag leaf opening and awns clearly visible—development point 2).

### Drought treatments

The plants were irrigated until the flag leaf appeared (Z37—Zadoks stage) and then subjected to two irrigation treatments: (i) well-watered treatment (abbreviated as C) in which the soil moisture was maintained at ~70% of field capacity (FC); and (ii) severe drought stress at 20% FC (abbreviated as D) following the methodology implemented by Kuczyńska et al. (2019). To maintain the targeted control and drought conditions, soil moisture in each pot was controlled gravimetrically by weighing and, if necessary, additionally volumetrically using the FOM/mts device (Ogrodowicz et al. 2017).

### Application of GA3 and trinexapac-ethyl under drought conditions

GA3 (10 mM, 100 mg/l) solution was sprayed directly onto the leaves at the beginning of tillering stage (Z21—Zadoks stage). The bioactive GA3 solution (Sigma-Aldrich) was prepared by dissolving the powder in distilled water. For each plant, 1–2 ml of GA3 solution was used. For the control plants, distilled water was sprayed. The GA3 treatment option was applied as described by Boden et al. (2014) with some modifications (treatment abbreviation D + GA). Trinexapac-ethyl (TR) was used as the commercial product Moddus 250 EC (Syngenta, USA) and was applied at the beginning of tillering stage (Z21). The TR treatment was applied according to the method of Grijalva-Contreras et al. (2012) with some modifications (treatment abbreviation D + TR).

### Phenotypic evaluation

In this study, phenological observations (developmental stages: tillering, flag_leaf, flowering, heading, maturity) as well as 14 yield-related traits were analyzed (Table 1).

**Table 1:** List of 14 phenotypic traits with description, abbreviations, and measured units

Trait (unit), abbrev.	Trait description
Total number of tillers, Tn	Number of tillers with fertile and non-fertile (without grains) spikes
Number of productive tillers, PTn	Number of tillers with fertile spikes
Length of main spike (cm), LSm	Length of main spike from 10 randomly selected spikes in a pot (without awns)
Number of spikelets per main spike, NSSm	Number of spikelets in spike of main stem—average for 10 main spikes in a pot
Number of grains per main spike, NGSm	Number of grains collected from one spike of main stem—average for 10 main spikes in a pot
Weight of gains per main spike (g), WGSm	Weight of grain collected from one spike of the main stem—average for 10 main spikes in a pot
Length of lateral spike (cm), LSl	Length of spike from lateral stem—average for 10 lateral spikes in a pot (without awns)
Number of spikelets per lateral spike, NSSl	Number of spikelets per spike of lateral stem—average for 10 lateral spikes in a pot
Number of grains per lateral spike, NGSl	Number of grains collected from spike of lateral stem—average for 10 lateral spikes in a pot
Weight of gains per main spike (g), WGSl	Weight of grain collected from one spike of the lateral stem—average for 10 lateral spikes in a pot
Grain yield (g), GY	Average weight of grains collected from one plant, calculated as average of measurements of grain weight for 10 plants
Thousand grain weight (g), TGW	Average weight of 1000 grains, calculated as average of 1000 * average weight of one grain for 20 spikes in a pot
Fertility of the main spike, FSm	NGSm/NSSm ratio
Fertility of the lateral spike, FSl	NGSl/NSSl ratio

### Evaluation of anther morphology

Anthers were measured using a stereomicroscope (Motic SMZ-161) following the protocol described by Browne et al. (2018) and were photographed at a fixed magnification using a digital camera system (Moticam CMOS BTU8). The photographs were studied using the Motic Advanced 3.2 software (Motic China Group Co., China). The length of the anthers (mm) was measured after they were removed from the primary flower of the largest spikelet. The width at their widest point was considered the width of the anthers (mm).

### Evaluation of pollen viability and morphology

Pollen viability and fertility were evaluated using TTC (2,3,5-triphenyl tetrazolium chloride) and KI/I2 (potassium iodide/iodine) staining methods as described by Ma et al. (2019) and Wang et al. (2012), respectively, with minor modifications. Anthers from different plants were used for each replicate. Pollen was extracted according to the protocol of Impe et al. (2020). First, fresh pollen was collected from the studied plants on the same day at 09:00. Then, pollen from each line was divided into two samples: one sample was incubated in a 1.5-ml centrifuge tube containing 0.1% TTC at 37 °C for 1 h, and the other was incubated in a 1.5-ml centrifuge tube containing 1% KI/I2 stain at room temperature for 5 min. The stained pollen was examined using a light microscope at a magnification of 400× (Motic BA410-E) and photographed using the Moticam digital camera. The images were analyzed using the Motic Advanced 3.2 software (Motic China Group Co., China). The pollen grains that had a round shape and were stained black with KI/I2 were classified as viable or alive, whereas those that were stained yellow or bright red were classified as sterile or dead. The pollen grains that stained red or pink with TTC were classified as viable (by the response to the presence of enzymatic activity), whereas those that appeared gray or colorless were considered sterile. A total of 2000 pollen grains were counted for each genotype in this study. Pollen viability for each genotype was then expressed as the percentage of the total number of live pollen grains to the total number of grains observed per field.

### Chlorophyll fluorescence measurements and OJIP test

Chlorophyll and fluorescence were measured on both control and stressed plants. Data were collected at two development points—LFE1 and LFE3, always at the same time of the day (09:00). Measurements were taken using a FluorPen FP 100-MAX (Photon Systems Instruments, Drasov, Czech Republic). Fluorescence transients for chlorophyll-a were recorded from the center of the completely spent leaf (second from the top). The leaves were dark-adapted for 30 min, before starting the measurements using leaf clips provided by the manufacturer. Then, the leaves were exposed to a pulse of saturating light at an intensity of 3000 μmol m^−2^ s^−1^ and all the studied parameters were measured. In each block, leaves from three plants of each line were measured. Nine replicates were performed for each cultivar and treatment (three leaves from three plants/treatment). The parameters used in this study to quantify the PSII behavior were as follows: the absorbed energy flux (ABS_RC), the trapped energy flux (TRo_RC), the electron transport flux (ETo_RC), the dissipated energy flux (DIo_RC), the maximum quantum yield of primary photochemistry (Fv_Fm), the probability/efficiency that a trapped exciton moves an electron in the electron transport chain beyond QA (Ψ_o), the quantum yield of the electron transport (Φ_Eo), the probability that the energy of an absorbed photon is dissipated as heat (Φ_Do), and the performance index (Pi_Abs).

### Genotyping

In this study, data from the Illumina 50K iSelect SNP array for barley were used to examine genomic similarities between the examined genotypes. The array included 44,040 working markers (Bayer et al. 2017). Further details on the genotyping procedure have been presented elsewhere (Mikołajczak et al. 2022). From the full set of markers, a subset of 23,747 markers polymorphic between the studied genotypes was selected.

### RT-qPCR analysis

Anthers were collected at two developmental stages (LFE1 and LFE3). Four biological replicates were collected for each stage, comprising approximately 100 anthers from four individual spikes. After dissection, the anthers were immediately frozen in liquid nitrogen and stored at −80 °C until RNA extraction was performed. The RNA was extracted using the RNeasy Mini Kit (QIAGEN, Germany) according to the manufacturer’s protocol with on-column DNase treatment (QIAGEN, Germany). Additionally, all isolated RNA samples were treated with TURBO DNase (Thermo Fisher Scientific, Lithuania) according to the manufacturer’s instructions to exclude trace contamination of samples with genomic DNA. The purity of all RNA samples was assessed via OD260/280 and OD260/230 absorbance ratios, whereas their structural integrity was evaluated using denaturing agarose gel electrophoresis. All RNA samples were adjusted to the same concentration (100 ng/μl). The quantitative real-time PCR (RT-qPCR) analysis performed in this study met the MIQE criteria (Bustin et al. 2009). Single-stranded cDNA was synthesized from 1 μg of total RNA using the iTaq Universal SYBR Green One-Step Kit according to the manufacturer’s instructions. To analyze the specific expression of each reference/target gene, RT-qPCR was performed using the CFX Connect Real-time PCR Detection System (Bio-Rad). Each 10 μl mixture for PCR contained 1 μl of a diluted RNA and 5 μM of each primer. To confirm the specificity of amplification and the absence of primer dimers, each run was completed with melting curve analysis (melting curve 63 to 95 °C, increasing by 0.5 °C for 0.05 s). Moreover, each pooled RT-qPCR product underwent sequencing process (AMU, Poznań, Poland). Data were normalized using three stable reference genes (UBI—GenBank ID: M60175.1; ACT1—GenBank ID: AY145451.1; UPL—GenBank ID: XM_045123725.1) and the stability of reference genes in the experimental setup was confirmed using a tool (Bio-Rad) that supports the geNorm algorithm. The RT-qPCR data for the genes and the endogenous controls were obtained from the means of three independent amplification reactions performed on four plants harvested at the same phenotypic stage (biological replicates). In each RT-qPCR run, extracts from the negative controls were applied. Gene expression data were analyzed using the Bio-Rad CFX Manager (Bio-Rad) software-CFX Maestro v2.0. Relative changes in the gene expression were calculated using the comparative 2^−ΔΔCt^ method and were normalized to the appropriate reference genes (Dawidziuk et al. 2014). *HvGAMYB* primers were designed using the Primer3 tool (https://primer3.org/). The complete list of primers and probes used is presented in Supplementary File 1.

### Statistical analysis

Kinship between genotypes was evaluated using Dice similarity coefficients computed from Illumina iSelect 50K SNP array data. The matrix of kinship estimates was used for hierarchical clustering of genotypes based on the average similarity algorithm and for principal coordinate analysis. Analysis of variance for observed quantitative traits was performed in the model containing fixed effects of groups of genotypes (early, late; G), treatments (T), and G × T interaction; significant effects were selected at *p* < 0.001 (approximate threshold resulting from application of the Bonferroni correction for multiple testing for all traits). In case of physiological parameters, due to non-normal distributions of observed variables, analysis of variance was performed on the data transformed by optimal Box-Cox transformation (Box and Cox, 1964). Grouping of experimental variants was done on the basis of Fisher’s protected least significant difference method at *p* < 0.05. Biplots were obtained using the principal component method. Pearson correlation coefficients were tested for significance based on *t* distribution. All statistical computations and visualizations were made in Genstat 22 (VSN International 2022).

## Results

### Classification of the genotypes

**Fig. 1:**
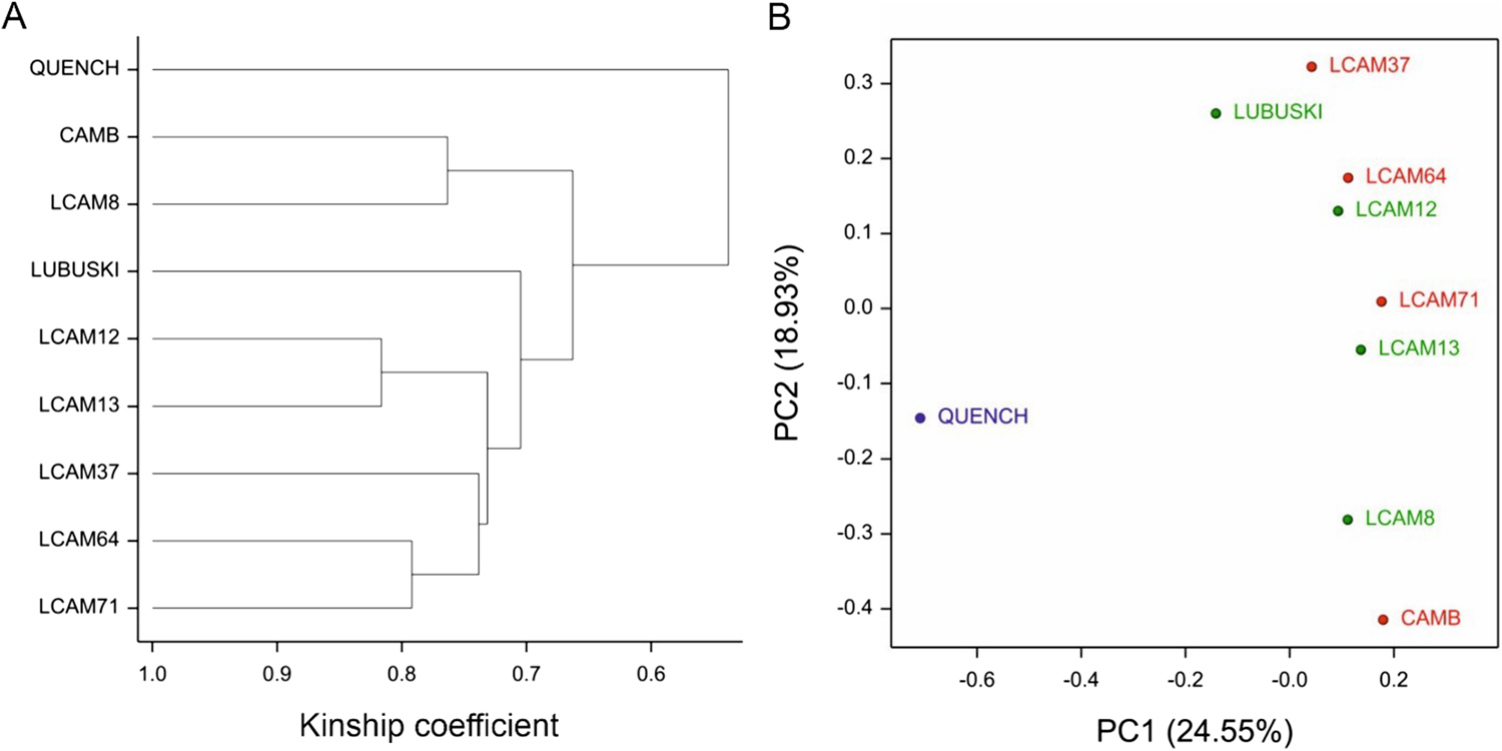
Relatedness of the studied genotypes based on kinship coefficients computed from Illumina iSelect 50K SNP array data. Dendrogram resulting from the cluster analysis (**A**). Genotypes in the system of first two axes resulting from principal coordinate analysis (PCoA) (**B**); red, early-heading genotypes; green, late-heading genotypes; blue, reference variety

Based on phenology observation and a previous study (Ogrodowicz et al. 2017), the plant material was divided into two subgroups: early-heading (CamB, LCam37, LCam64, and LCam71) and late-heading (Lubuski, LCam08, LCam12, and LCam13) genotypes. The genetic similarity of the studied barley genotypes was analyzed using hierarchical clustering and principal coordinate analysis (PCoA) (Fig. 1). The genotypes of the plants clustered mainly according to their heading time: plants classified as early-heading genotypes clustered together.

### Plant phenology under stress conditions

**Fig. 2:**
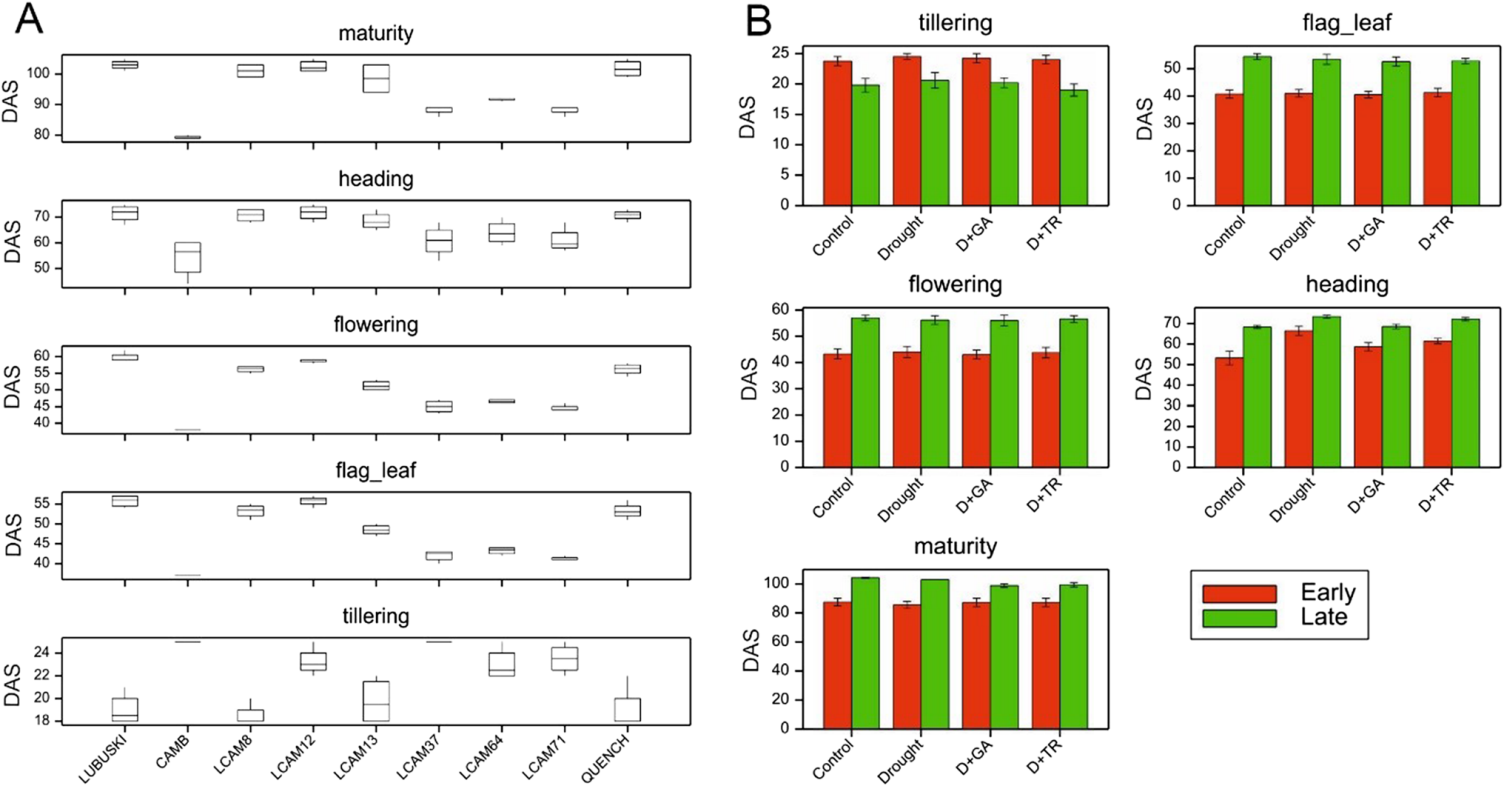
Distribution of phenological observations for the studied genotypes (**A**). Mean values (with standard errors) of phenological traits for the studied genotypes grown under four treatments: control, drought, D + GA (combination of drought condition and GA3 foliar spray), and D + TR (combination of drought condition and trinexapac-ethyl foliar spray) (**B**). Early, early-heading group of studied plants; Late, late-heading group of studied plants. DAS, days after sowing

The mean values for all studied traits are presented in Supplementary File 2. The results of the analysis of variance (ANOVA) showed significant differences (at *p* < 0.001) between groups of genotypes for all phenological traits and a significant effect of the applied treatments only on heading (Supplementary File 3). The influence of the treatments in the heading stages (Fig. 2B) was such that the drought conditions delayed the plant development for both early- and late-heading genotypes. Under D + GA conditions, acceleration of plant development was observed in both plant subgroups compared with the drought conditions. The D + TR conditions resulted in the acceleration of development compared with the drought conditions in the early-heading genotypes group.

### Evaluation of yield-related traits

The results of ANOVA revealed the significant effects of the applied treatments on all the investigated yield-related traits (Supplementary File 3). Significant differences between groups of genotypes were also observed, as well as effects of G × T interaction for the vast majority of the studied traits. The mean values of most of the studied traits showed significant reduction under drought conditions with some exceptions: significant increases in Tn and PTn was observed in both early- and late-heading plants compared to C condition (Supplementary File 4).

An increase in Tn was also observed under D + GA condition (compared to C condition) in the late-heading plants. In this conditions, the early-heading plants showed Tn mean values similar to those under control condition. For traits related to the lateral spike (LSl, NSSl, NGSl, and WGSl), drought conditions generally resulted in a significant decrease in mean values, with a few exceptions, such as the mean value of NSSl in the late-heading plants grown under D and D + TR conditions. The early- and late-heading plants differed in terms of main spike fertility recorded under C and D conditions (Supplementary File 4). However, no significant differences were found in FSl between early- and late-heading plants grown under all types of applied conditions (an exception: D condition). The results of ANOVA showed a significant effect for FSm in relation to treatments and T × G.

**Fig. 3:**
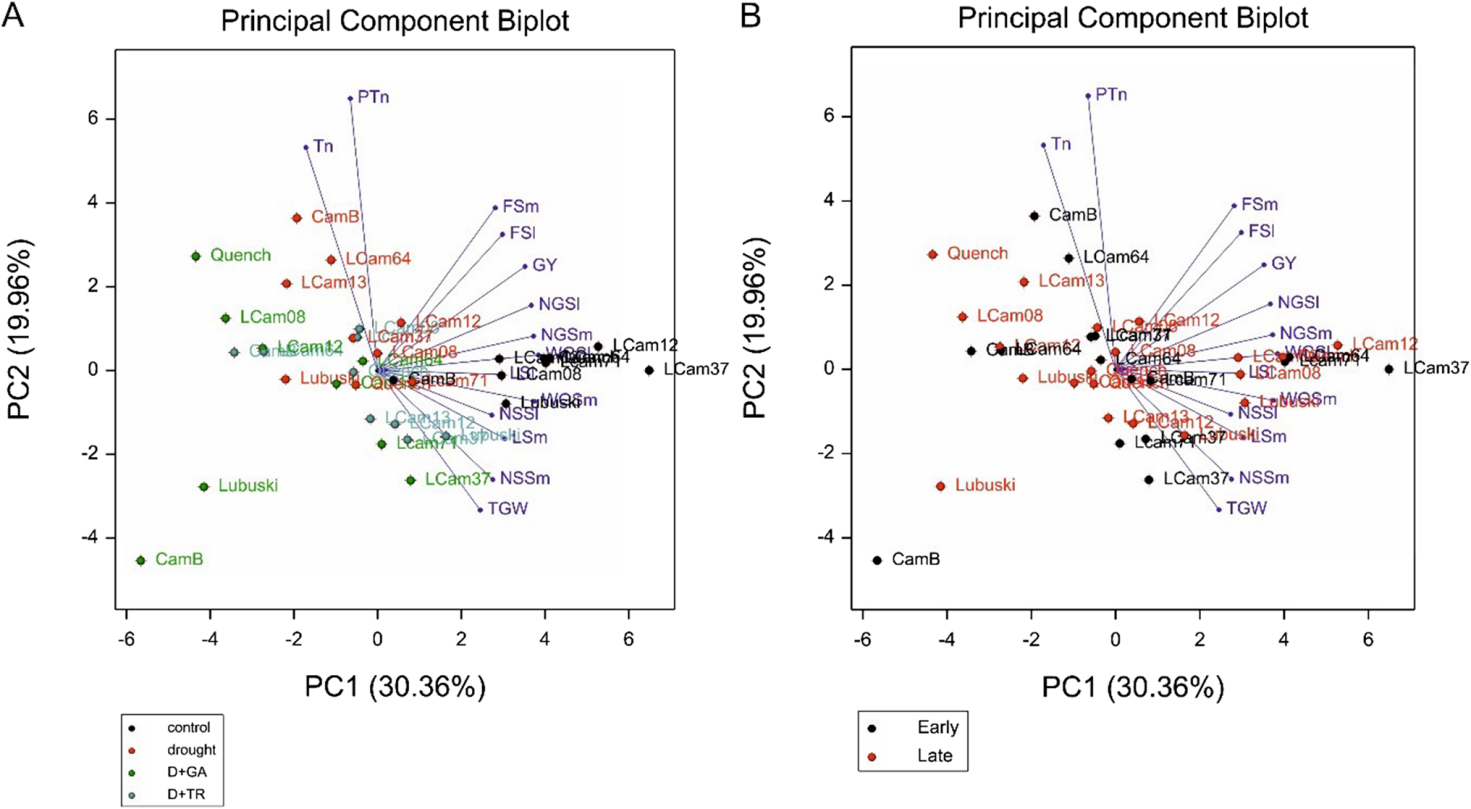
Biplot visualization of variability of yield-related traits between applied treatments—control, drought, D + GA (combination of drought condition and GA3 foliar spray), and D + TR (combination of drought condition and trinexapac-ethyl foliar spray) (**A**) and division on groups of early- and late-heading genotypes (**B**)

Principal component analysis (PCA) was used to visualize the variability of yield-related traits in two variants: to show differences between treatments and between earliness groups. Plants grown under C conditions were distributed on the right side of the plot in close proximity to each other which shows low variability, especially with respect to tillering traits correlated with PC2 (Fig. 3A). Figure 3A also shows that the accessions had a long projection on the vectors associated with the yield-related traits such as NGSm, WGSl, and WGSm. The use of additional growth regulators (GA and TR) resulted in a shift of genotypes in the biplot. In the D + GA treatment, the plants were largely dispersed, with the parental genotypes on the left side of the plot. The studied genotypes were not discriminated into subgroups corresponding with their developmental patterns (early- and late-heading plants) (Fig. 3B).

The correlation coefficients recorded between studied yield-related are shown in Supplementary File 5.

### OJIP parameter analysis

The effects of treatment on the majority of the studied parameters were significant at LFE3, whereas significant effects of treatment were found in two cases at LFE1 (Ψ_o and Φ_Eo). A significant difference was observed for ABS_RC at LFE1 between early- and late-heading subgroups of plants in all types of the applied treatments (an exception: C condition) (Supplementary File 6). At LFE3, the ABS_RC differences between the early- and late-heading accessions were observed under control, D + GA, and D + TR conditions. Dlo_RC was affected by all the applied treatments both at LFE1 and LFE3, and differences were observed between the subgroups (except under D + TR conditions at LFE3). The applied stress conditions affected Fv_Fm, and differences were detected between the plant subgroups (except under D + TR conditions at LFE3). At the second time of measurement, the significant Pi_Abs reduction in mean values was recorded for both studied subgroups.

**Fig. 4:**
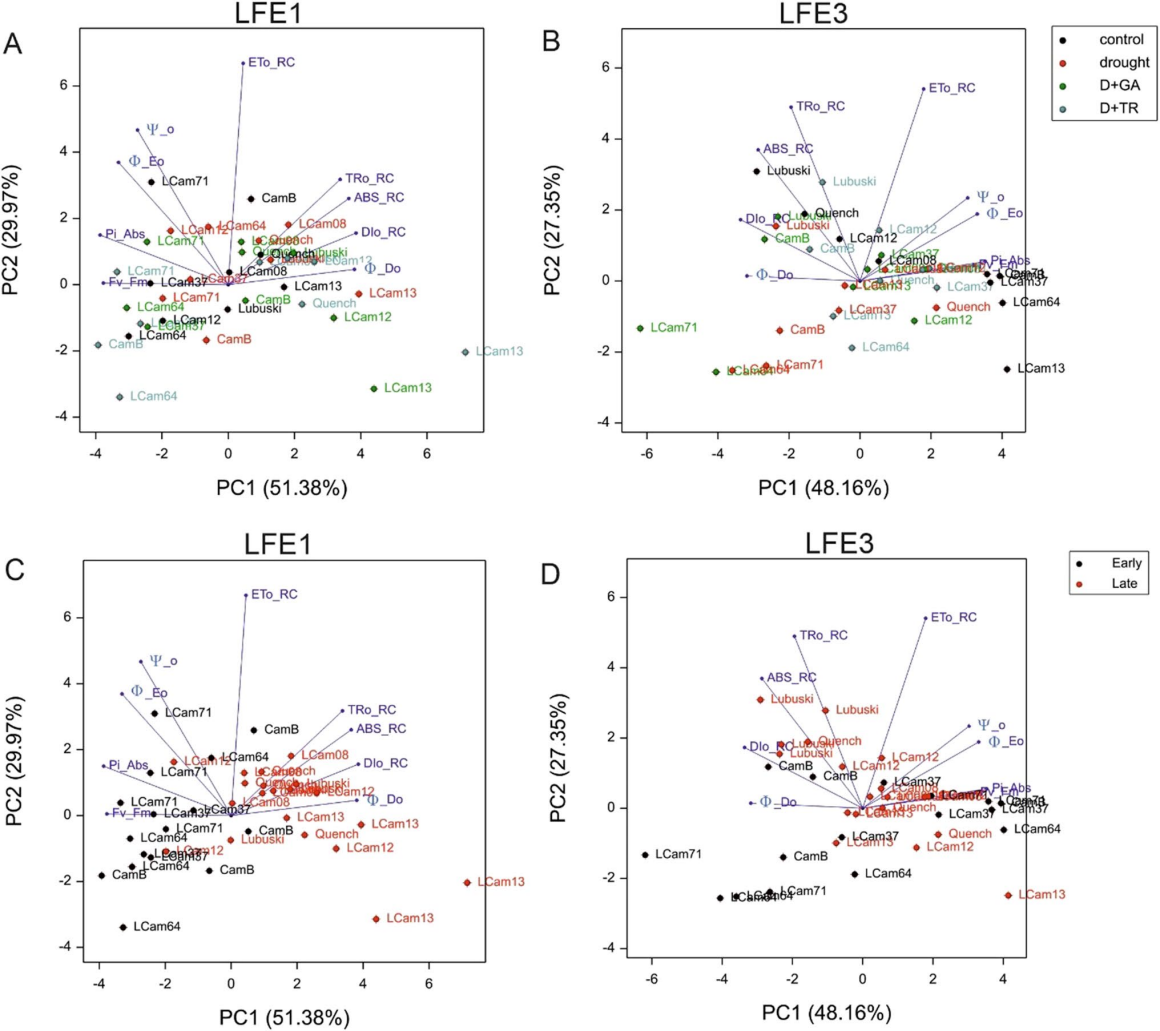
Biplot visualization of variability of OJIP parameters (measured in two development points—LFE1 and LFE3) between applied treatments—control, drought, D + GA (combination of drought condition and GA3 foliar spray), and D + TR (combination of drought condition and trinexapac-ethyl foliar spray) (**A**, **B**) and groups of early- and late-heading genotypes (**C**, **D**)

Figure 4 visualizes the variability of OJIP parameters in the studied accessions, with respect to the applied treatments (Fig. 4A, B) and the division between the two subgroups of plants (Fig. 4C, D). On the first case, random distribution of the studied genotypes was observed, whereas in the second, the plant material is grouped in terms of earliness. In the first measurement (Fig. 4C), the early-heading genotypes were located on the left side of the plot, whereas the late-heading plants were on the right side (with some exceptions). This pattern was not visible in the second measurement of the fluorescence parameters (Fig. 4D). The correlation coefficients between studied OJIP parameters are shown in Supplementary File 7.

### Anther morphology evaluation

**Fig. 5:**
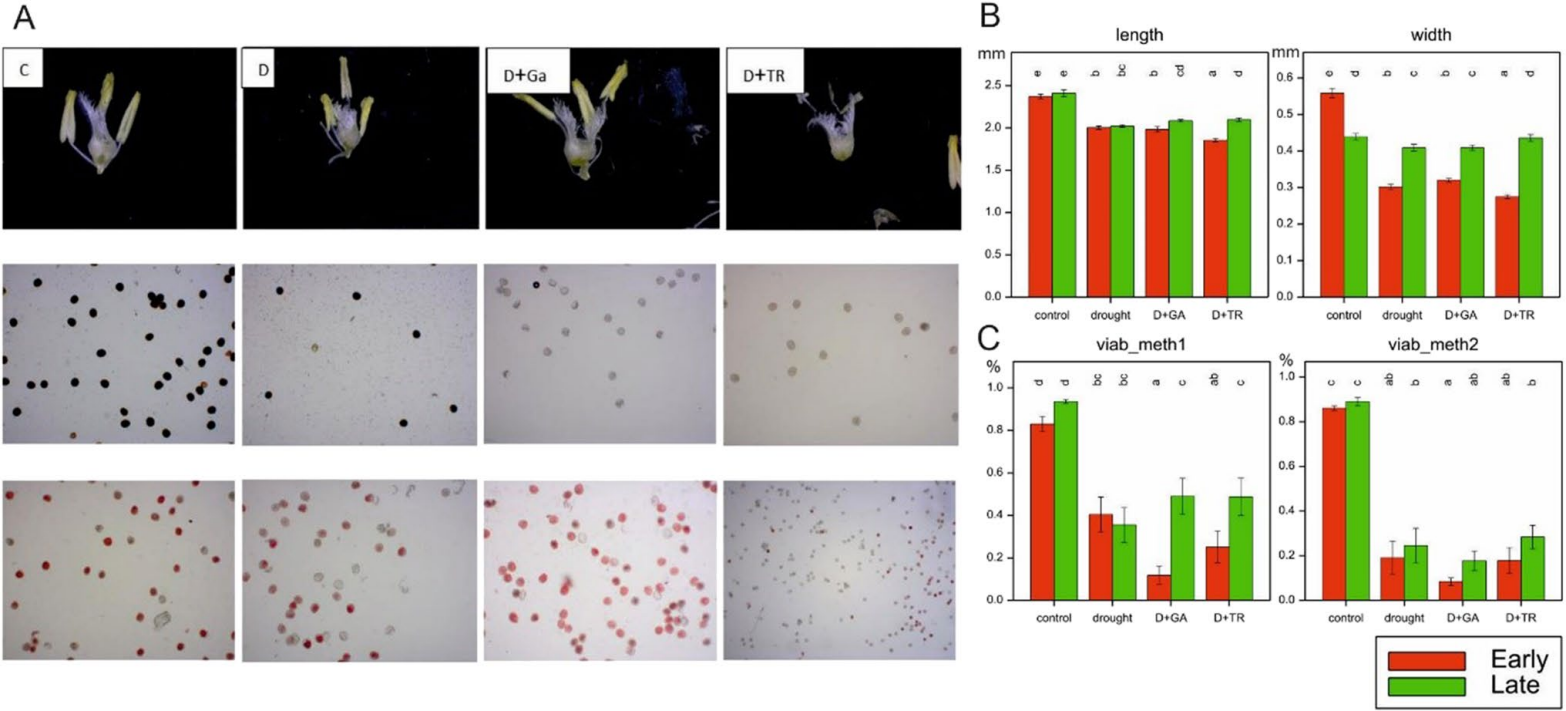
Comparison of pollen viability photographed by light microscopy and evaluated using two different staining methods in four different treatments (**A**), length and width of the anthers dissected from the two subgroups of plants subjected to four different treatments (**B**), and pollen viability evaluation using two different staining methods (**C**). Treatments: control, drought, D + GA (combination of drought condition and GA3 foliar spray), and D + TR (combination of drought condition and trinexapac-ethyl foliar spray). Early, early-heading group of studied plants; Late, late-heading group of studied plants. Letters indicate statistically similar treatments or plant groups at *p* < 0.05 according to the Fisher least significant difference test

The results of ANOVA showed significant effects of treatment, subgroups, and of T × G interaction on anther length and width (Supplementary File 3). Under control conditions, no significant differences in anther lengths were observed between the two subgroups of plants (Fig. 5B). All applied stress conditions affected the anther length of the studied genotypes. Anther width differed between the two plant subgroups under control conditions, and the applied stress treatments had an impact on the reduction in the anther width of the early-heading plants. The reduction in this trait of this magnitude was not observed in the late-heading plants.

### Pollen viability evaluation

The results of ANOVA showed significant effects of treatments on pollen viability using two different staining methods (Supplementary File 3, Fig. 5A). Comparison of pollen viability between the two subgroups of plants using method 1 showed no significant differences under control conditions. Applied stressed conditions caused the reduction of pollen viability evaluated by method 1. The lowest mean values of this trait were observed for early-heading genotypes under D + GA conditions, whereas in the late-heading plants, a slight increase in pollen viability using method 1 was observed in these treatments compared with drought conditions. A rapid decrease in pollen viability estimated using method 2 was observed for the studied plants under stress conditions (a greater decrease in this trait was observed for the early-heading plants) (Fig. 5C).

### HvGAMYB transcript level

**Fig. 6:**
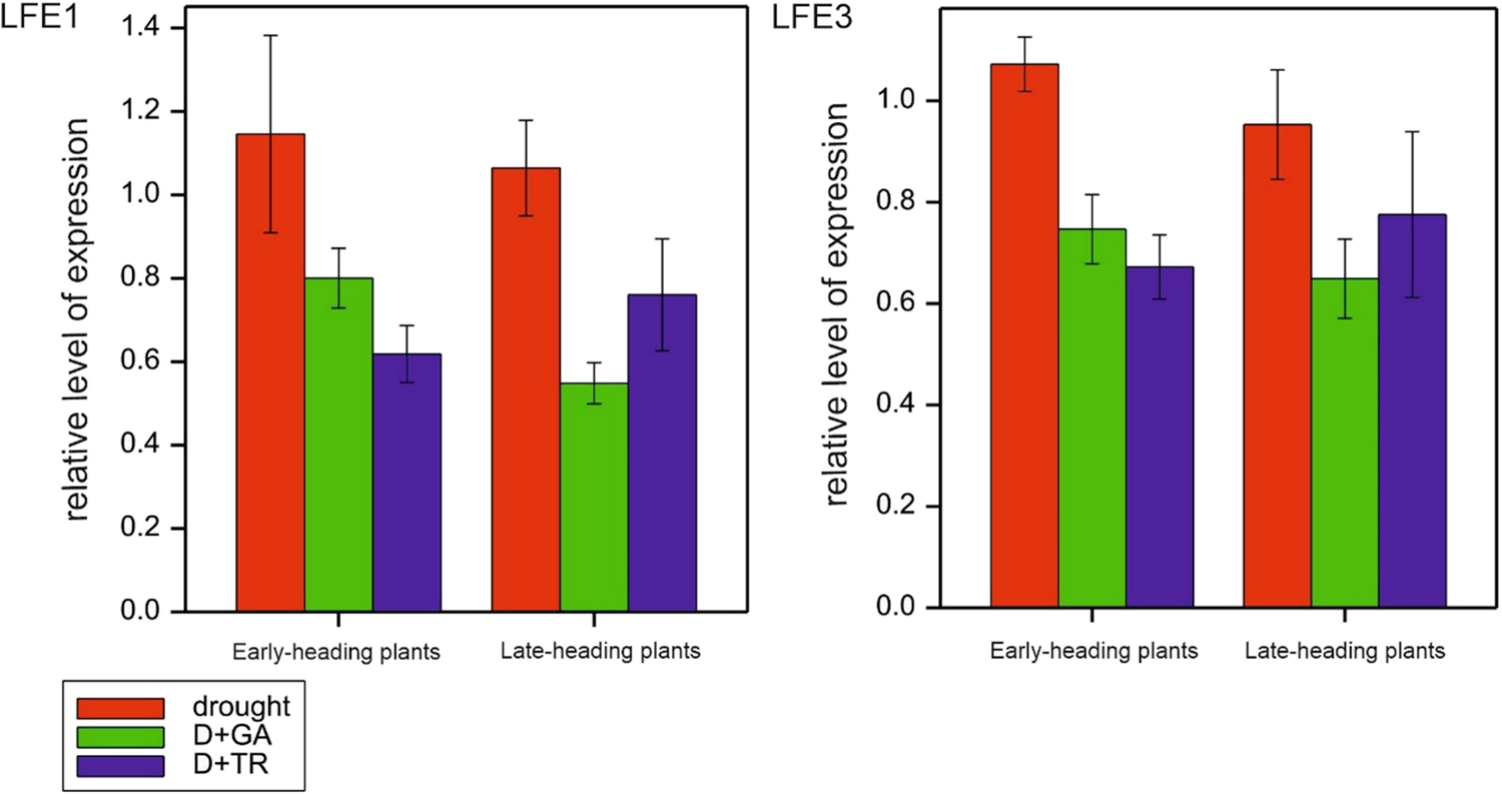
Analysis of the expression pattern of *HvGAMYB* in plant tissues collected from barley genotypes varied in terms of phenology (mean values of relative level expression from early-heading plants CamB, LCam37, LCam64, and LCam71 and late-heading plants Lubuski, LCam08, LCam12, and LCam13). The tissue probes were collected in two development points (LFE1 and LFE3). Treatments: drought, D + GA (combination of drought condition and GA3 foliar spray), and D + TR (combination of drought condition and trinexapac-ethyl foliar spray)

*HvGAMYB* expression was analyzed at two development points and presented as the mean values of relative level of expression from early-heading plants CamB, LCam37, LCam64, and LCam71 and late-heading plants Lubuski, LCam08, LCam12, and LCam13. The results of ANOVA showed significant effects of treatments at LFE1 (*p* < 0.001) on the expression of the studied gene. Under drought conditions, a slight decrease in gene relative expression was observed for both plant subgroups at LFE3 compared with LFE1 (Fig. 6).

Under D + GA conditions, the early-heading plants exhibited higher levels of gene expression at LFE1 and LFE3 (compared to the late-heading plants), but the decrease in *HvGAMYB* expression for the early-heading subgroup at LFE3 was still observed compared with LFE1. For the late-heading plants, an increase in the studied gene expression level was noticed at LFE3 compared with LFE1 in this type of treatment. Under D + TR conditions, gene expression was lower in the early-heading plants than in the late-heading plants for both times of gene expression level measurements. At LFE3, *HvGAMYB* relative expression increased in the early-heading plants under this condition compared with this gene expression level recorded at LFE1.

**Fig. 7:**
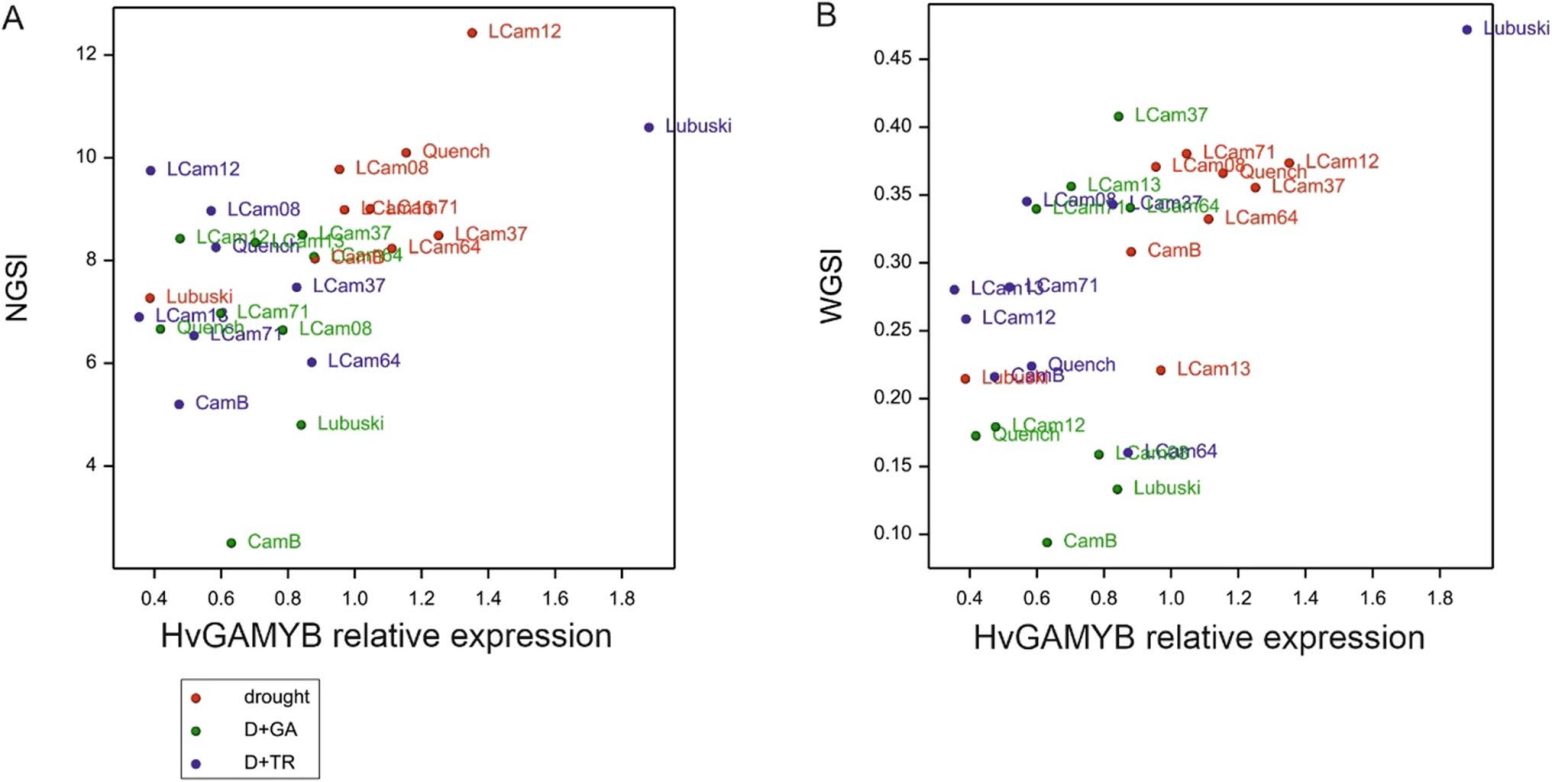
Scatterplots of NGSl (**A**) and WGSl (**B**) versus relative level of expression of *HvGAMYB* measured at development point 2 (LFE3). Treatments: drought, D + GA (combination of drought condition and GA3 foliar spray), and D + TR (combination of drought condition and trinexapac-ethyl foliar spray). NGSl, number of grains per lateral spike; WGSl, weight of grains per lateral spike (g). Early-heading group: CamB, LCam37, LCam64, and LCam71; late-heading group: Lubuski, LCam08, LCam12, and LCam13

*HvGAMYB* expression at development point 2 was positively correlated with NGSl (*r*^2^ = 0.53; *p* = 0.004) and WGSl (*r*^2^ = 0.59; *p* = 0.002). The highest relative gene expression for Lubuski under D + TR condition was accompanied by the highest value of WGSl. The relationship between NGSl, WGSl, and *HvGAMYB* expression level at development point 2 is presented on Fig. 7. Greater values of *HvGAMYB* expression level and NGSl were recorded for plants subjected to drought condition compared to plants grown under artificial development modifications (GA and TR) combined with drought.

## Discussion

In this study, artificial growth regulators (exogenous GA3 and the GA inhibitor—TR) were employed to highlight the effects of growth-type habits on the reaction of plants to drought stress. The classification of the studied plants into two subgroups in terms of phenology allowed us to investigate the response of plants with different heading time to drought stress. In the present study, morphological and phenological observations were confirmed by genotyping data because plant division based on the genetic profiles overlapped with the type of growth habits.

For the vast majority of the studied yield-related traits, significant effects were recorded for treatment, group, and T × G. According to previous studies (Wu et al. 2007; Kottmann et al. 2016), plants with different types of growth habits react in different ways to most of the abiotic stresses, which was confirmed also in this investigation. For the early- and late-heading plants, the morphology and ability to adapt to conditions varied significantly and heading time was the important factor contributing to direct stress responses (Shavrukov et al. 2017). For example, in the present study, under drought conditions, the plants developed more tillers compared with control conditions. Interestingly, the early-heading plants grown under drought condition were characterized by higher mean values of productive tillers compared with the late-heading plants, which suggests that the development of tillers with fertile spikes was the main goal of the plant’s strategy to survive (to distribute the progeny) under unfavorable conditions. This finding is in line with previous studies (Mosaad et al. 1995; Xie et al. 2016; Moeller and Rebetzke 2017). The acceleration of plant development (using external GA application) disturbed this strategy: no rapid tiller development was observed under D + GA conditions for the early-heading plants, which may be associated with the changes in plant growth. Under D conditions, the early-heading genotypes showed rapid development and—probably—shaped the tillers only in the secondary tillering process in the rewatering phase. These findings are in line with the nature of tiller development, which is considered a plastic process, being strongly dependent on environmental factors that may promote, or repress, lateral shoot development through a complex network of hormonal and regulatory signals (Kebrom et al. 2012). ANOVA showed that for a trait linked to spike fertility (FSm), significant effects were recorded for treatments and G × T interaction, which suggests that an appropriate seed development process may be associated with the right growth strategy under drought conditions. This finding is in agreement with that of a previous study (Begum et al. 2022). Successful development of anthers in flowering plants is crucial because plant fertility and, as a consequence, yield productivity depend on delivering the male gamete to the female gametophyte for efficient fertilization (Borg et al. 2009). However, anther development is often perturbed by abiotic stresses such as drought, resulting in male sterility and yield reduction (Jin et al. 2013).

The results of the PCA of the yield-related traits of the studied plants revealed that the genotypes clustered in close proximity to each other in terms of the applied treatment (control and drought conditions), but the locations of the studied plants were disrupted when drought conditions combined with foliar growth regulators were applied. This finding shows that the studied plants exhibited different responses to plant growth modifications (GA and TR growth regulators) under drought conditions and, as a consequence, showed different yield performances.

In many studies, chlorophyll fluorescence has long been used as a convenient and sensitive indicator of plant stress responses (e.g., Goltsev et al. 2005; Kalaji et al. 2016). Fluorescence increase or induction curves, usually called the OJIP test, have been also adapted for screening different varieties of crops subjected to drought stress (Yao et al. 2018), including barley (Daszkowska-Golec et al. 2019). Seleiman et al. (2021) suggested that drought stress is a complicated stressor and that different aspects of plant growth and physiology should be taken into account for the evaluation of plants’ response to drought stress. Therefore, in the present study, the exploration of plants’ response to drought was complemented with both yield performance and physiological analyses. Under stress conditions, significant increases in the parameters linked to specific energy fluxes per reaction center (RC) (e.g., ABS_RC) were recorded. In the present study, significant differences were observed for ABS_RC at LFE1 between two subgroups of plants in all types of stress treatments. It is worth noting that much lower ABS_RC values were recorded for the early-heading plants during the first measurement, but over time (LFE3), the mean values observed for this trait were almost similar for both plant subgroups, which emphasizes the role of stress duration in plants’ response to unfavorable conditions. According to Jedmowski and Brüggemann (2015), inactivation of some RCs increases the ABS_RC under drought stress conditions. Another reason for the increase in ABS_RC is degradation of chlorophyll through early leaf senescence induced by drought stress (Boureima et al. 2012) or regrouping of antennae from inactive PSII RCs to active (Kalaji et al. 2016). Changes in ABS_RC recorded in the present study may suggest that the early-heading genotypes react differently to the initial phase of drought stress, but after a while, damage to RCs occurs also in this type of plant. Since the excess of energy unutilized in photochemistry is dissipated as heat, the increase of DIo_RC was expected. The mean values of this parameter monitored in studied plants changed depending on the development points of measurements (the increase in DIo_RC mean values was observed after prolonged drought stress, especially in early-heading plants). This results highlighted the diversity of stress response observed in plants varied in terms of phenology. The decrease in quantum efficiency (Ψ_o, Pi_Abs) and the increase in heat dissipation (indicated by DIo_RC and Φ_Do) were observed in the present study. These findings are in line with the studies that investigated wheat (*Triticum aestivum* L.) plants subjected to combination of drought and heat stresses (Zhu et al. 2021) and tomato (*Solanum lycopersicum* L.) plants grown under drought condition (Sousaraei et al. 2021), who found a rapid increase in parameters DIo_RC, Φ_Do, and TRo_RC and a decrease in Pi_Abs, Fv_Fm, and Eto_RC mean values. In this study, the random distribution of genotypes on the biplot presenting variability of OJIP parameters in LFE3 showed that prolonged drought stress caused also that among early- and late-heading subgroups, the studied plants exhibited different responses to applied stress conditions.

Developmental defects in the tapetum and a lack of starch accumulation are caused by water-deficit stress in pollen grains (Nguyen et al. 2009; Ji et al. 2010), which was confirmed in our investigation as viability monitoring by method 1 decreases significantly under stressed conditions. Stress-tolerant wheat cultivars can maintain starch accumulation and sink strength during the young microspore stage under water stress conditions (Ji et al. 2010), which was not confirmed in our study, as there were no differences in pollen viability evaluated by method 1 (JKJ method) between the early- and late-heading plants under D conditions. The artificial acceleration of the growth of the early-heading plants contributed to the impairment of pollen viability, exacerbating the negative impact of drought on pollen development.

In many plant tissues (barley aleurone, wheat internodes, and anthers), *GAMYB* expression has been shown to be directly upregulated by the gibberellin—GA3 (Gubler et al. 1995). In the present study, *HvGAMYB* expression was confirmed in the anther tissues of plants subjected to different water conditions. It is interesting to note that depending on the applied growth regulators, *HvGAMYB* relative expression level was different for the early- and late-heading plants. Transgenic barley lines with an excess of fourfold levels of endogenous GAMYB protein in their anthers were reported to be male sterile (Murray et al. 2003; Duca et al. 2008). Also, a progressive decrease in anther size was associated with the increase in *GAMYB* levels, particularly a decrease in anther length (Murray et al. 2003). These findings are in line with results obtained in the present study, where for the early-heading plants with a higher *HvGAMYB* expression level, lower mean values of trait linked to anther morphology (anther width) were recorded compared to the late-heading plants. In our study, *HvGAMYB* was expressed at a relatively high level both in anthers collected from the early- and late-heading plants subjected to drought. Interestingly, under D conditions with the growth regulator applications, the relative expression levels of *HvGAMYB* were lower compared to these recorded under drought condition without spraying interactions, which highlighted the complex nature of some TFs expression fluctuation in terms of plant growth modifications under drought condition. In our study, the application of exogenous GA3 spry did not increase the *HvGAMYB* expression—contrary to expectation. This can be associated with the fact that the *HvGAMYB* transcripts could be unstable and these differences in *HvGAMYB* expression levels between treatments (D and D + GA) could be related to the time of GA3 application (tillering stage). On the other hand, for the early-heading plants, still a higher level of *HvGAMYB* expression under D + GA conditions was demonstrated, which confirms the association of the studied gene with early flowering and GA signaling. In the study conducted by Murray et al. (2003), strong *HvGAMYB* over-expresser plants were male sterile. Although pollen from over-expressers was smaller and more irregular than that from null segregants, pollen development (with respect to starch accumulation) appeared to progress normally in male sterile anthers. However, in our study, the pollen of early-heading plants evaluated by method 1 (staining starch with Lugol’s iodine solution) was much less viable under D + GA condition (compared to the late-heading plants).

The results of this study also show that the *HvGAMYB* expression level evaluated at development point 2 is correlated positively with traits associated with lateral spike morphology (NGSl and WGSl). This finding suggests that the mentioned transcription factor could have an important role in yield formation under drought condition—especially in terms of lateral spikes development—but further investigation is needed to fully explore the complex nature of pollen development where GA signal transduction pathway may be modified by a wide range of internal and external factors.

## Supplementary information

Supplementary File 1 DNA sequences of the gene specific primers (DOCX 14 kb)

Supplementary File 2 Mean values for studied traits (XLSX 46 kb)

Supplementary File 3 Results of analysis of variance for observed traits (P values for testing significance of variation sources) (DOCX 15 kb)

Supplementary File 4 Distribution of mean yield-related trait values. Early: early-heading group of studied plants, Late: late-heading group of studied plants. Letters indicate statistically similar treatments or plant groups at p < 0.05 according to the Fisher least significant difference test (DOCX 422 kb)

Supplementary File 5 Correlations between the studied traits of genotypes differentiated in terms of phenology (p < 0.01) (DOCX 14 kb)

Supplementary File 6 Results of measurements of OJIP at LFE1 (A) and LFE3 (B). Mean values shown in the optimal Box-Cox transformation. Early: early-heading group of studied plants, Late: late-heading group of studied plants. Letters indicate statistically similar treatments or plant groups at p < 0.05 according to the Fisher least significant difference test (DOCX 281 kb)

Supplementary File 7 Correlations between the studied OJIP parameters of genotypes differentiated in terms of phenology (p value < 0.01) recorded at development point 1 (A), development point 2 (B) (DOCX 24 kb)
