# Supplementary material for: The effects of heading time on yield performance and HvGAMYB expression in spring barley subjected to drought
Source: J Appl Genet. 2023 Mar 10;64(2):289–302. doi: 10.1007/s13353-023-00755-x (PMC10076406; doi:10.1007/s13353-023-00755-x)
Supplement: Supplementary file 1 — DNA sequences of the gene specific primers (DOCX 14 kb) [file 13353_2023_755_MOESM1_ESM.docx]

The effects of heading time on yield performance and *HvGAMYB* expression in spring barley subjected to drought

Piotr Ogrodowicz*, Anetta Kuczyńska, Paweł Krajewski, Michał Kempa

Institute of Plant Genetics of the Polish Academy of Sciences, Strzeszyńska 34, 60-479 Poznań, Poland

*Corresponding authors:

Tel.: (+48 61) 65 50 224; e-mail: pogr@igr.poznan.pl

| Gene | Sequence (5′→3′) | Ampl. length (bp) | Melt temp.  [ºC] | E [%] |
| --- | --- | --- | --- | --- |
| *HvGAMYB* | F: CCTTCACTCCAAGATACCGAAT | 89 | 63 | 108.3 |
|  | R: GGATCAACCAACTCCGTAGG |  | 63 |  |
| *ACT1* | F: GCCGTGCTTTCCCTCTATG | 235 | 63 | 101.8 |
|  | R: GCTTCTCCTTGATGTCCCTTA |  | 63 |  |
| *UBI* | F: TCGCCGTCCTCCAGTTCTAC | 63 | 63 | 105.1 |
|  | R: CCTTCCTGAGCCTGGTTACCT |  | 63 |  |
| *UPL* | F: CTGAAGAGTTAGGCGGGAAA | 100 | 63 | 105.8 |
|  | R: ATCGCATGAACGTAGTGCAA |  | 63 |  |

Supplementary File 1. DNA sequences of the gene specific primers
