# Supplementary material for: The effects of heading time on yield performance and HvGAMYB expression in spring barley subjected to drought
Source: J Appl Genet. 2023 Mar 10;64(2):289–302. doi: 10.1007/s13353-023-00755-x (PMC10076406; doi:10.1007/s13353-023-00755-x)
Supplement: Supplementary file 3 — Results of analysis of variance for observed traits (P values for testing significance of variation sources) (DOCX 15 kb) [file 13353_2023_755_MOESM3_ESM.docx]

The effects of heading time on yield performance and *HvGAMYB* expression in spring barley subjected to drought

Piotr Ogrodowicz*, Anetta Kuczyńska, Paweł Krajewski, Michał Kempa

Institute of Plant Genetics of the Polish Academy of Sciences, Strzeszyńska 34, 60-479 Poznań, Poland

*Corresponding authors:

Tel.: (+48 61) 65 50 224; e-mail: pogr@igr.poznan.pl

| Group of traits | Trait |  | | |
| --- | --- | --- | --- | --- |
|  |  | Group of genotypes (G) | Treatment (T) | G × T interaction |
| Phenology | Tillering | < 0.001 | 0.665 | 0.930 |
|  | Flag leaf | < 0.001 | 0.893 | 0.905 |
|  | Flowering | < 0.001 | 0.976 | 0.997 |
|  | Heading | < 0.001 | < 0.001 | 0.103 |
|  | Maturity | < 0.001 | 0.341 | 0.278 |
| Phenotype | Tn | 0.001 | < 0.001 | < 0.001 |
|  | PTn | 0.015 | < 0.001 | < 0.001 |
|  | LSm | < 0.001 | < 0.001 | < 0.001 |
|  | NSSm | < 0.001 | < 0.001 | < 0.001 |
|  | NGSm | 0.001 | < 0.001 | < 0.001 |
|  | WGSm | 0.093 | < 0.001 | < 0.001 |
|  | LSl | 0.432 | < 0.001 | 0.001 |
|  | NSSl | 0.004 | < 0.001 | < 0.001 |
|  | NGSl | 0.026 | < 0.001 | 0.001 |
|  | WGSl | 0.004 | < 0.001 | < 0.001 |
|  | GY | < 0.001 | < 0.001 | 0.003 |
|  | TGW | < 0.001 | < 0.001 | < 0.001 |
|  | FSm | 0.770 | < 0.001 | < 0.001 |
|  | FSl | 0.661 | < 0.001 | 0.048 |
| Chlorophyll fluorescence, LFE1 | ABS_RC | < 0.001 | 0.067 | < 0.001 |
|  | TRo_RC | < 0.001 | 0.092 | < 0.001 |
|  | ETo_RC | 0.007 | 0.005 | 0.004 |
|  | DIo_RC | < 0.001 | 0.056 | < 0.001 |
|  | Fv_Fm | < 0.001 | 0.122 | < 0.001 |
|  | Ψ_o | < 0.001 | < 0.001 | 0.007 |
|  | Φ_Eo | < 0.001 | < 0.001 | 0.006 |
|  | Φ_Do | < 0.001 | 0.098 | < 0.001 |
|  | Pi_Abs | < 0.001 | 0.009 | < 0.001 |
| Chlorophyll fluorescence, LFE3 | ABS_RC | 0.003 | 0.001 | < 0.001 |
|  | TRo_RC | < 0.001 | 0.012 | 0.011 |
|  | ETo_RC | < 0.001 | < 0.001 | < 0.001 |
|  | DIo_RC | 0.141 | < 0.001 | < 0.001 |
|  | Fv_Fm | < 0.001 | < 0.001 | < 0.001 |
|  | Ψ_o | 0.951 | < 0.001 | < 0.001 |
|  | Φ_Eo | 0.308 | < 0.001 | < 0.001 |
|  | Φ_Do | < 0.001 | 0.002 | < 0.001 |
|  | Pi_Abs | 0.572 | < 0.001 | < 0.001 |
| Anther morphology | Length | < 0.001 | < 0.001 | < 0.001 |
|  | Width | < 0.001 | < 0.001 | < 0.001 |
|  |  |  |  |  |
| Pollen viability | Method 1 | 0.001 | < 0.001 | 0.023 |
|  | Method 2 | 0.054 | < 0.001 | 0.869 |
| GAMYB relative expression | LFE1 | 0.542 | < 0.001 | 0.294 |
|  | LFE3 | 0.631 | < 0.004 | < 0.479 |

Supplementary File 3. Results of analysis of variance for observed traits (P values for testing significance of variation sources)
