# Supplementary material for: The effects of heading time on yield performance and HvGAMYB expression in spring barley subjected to drought
Source: J Appl Genet. 2023 Mar 10;64(2):289–302. doi: 10.1007/s13353-023-00755-x (PMC10076406; doi:10.1007/s13353-023-00755-x)
Supplement: Supplementary file 4 — Distribution of mean yield-related trait values. Early: early-heading group of studied plants, Late: late-heading group of studied plants. Letters indicate statistically similar treatments or plant groups at p < 0.05 according to the Fisher least significant difference test (DOCX 422 kb) [file 13353_2023_755_MOESM4_ESM.docx]

The effects of heading time on yield performance and *HvGAMYB* expression in spring barley subjected to drought

Piotr Ogrodowicz*, Anetta Kuczyńska, Paweł Krajewski, Michał Kempa

Institute of Plant Genetics of the Polish Academy of Sciences, Strzeszyńska 34, 60-479 Poznań, Poland

*Corresponding authors:

Tel.: (+48 61) 65 50 224; e-mail: pogr@igr.poznan.pl


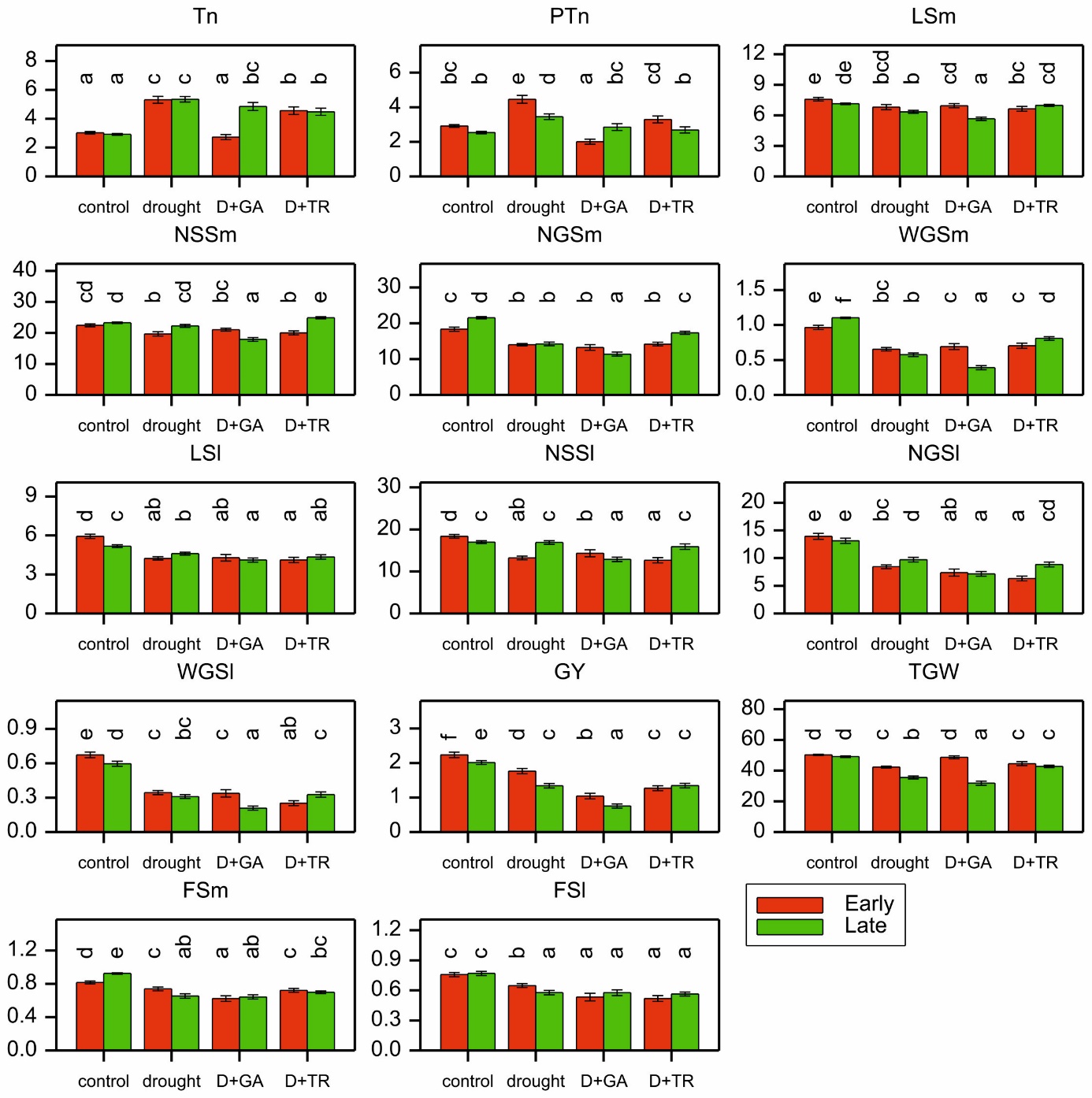


Supplementary File 4. Distribution of mean yield-related trait values. Early: early-heading group of studied plants, Late: late-heading group of studied plants. Letters indicate statistically similar treatments or plant groups at *p* < 0.05 according to the Fisher least significant difference test
