# Supplementary material for: The effects of heading time on yield performance and HvGAMYB expression in spring barley subjected to drought
Source: J Appl Genet. 2023 Mar 10;64(2):289–302. doi: 10.1007/s13353-023-00755-x (PMC10076406; doi:10.1007/s13353-023-00755-x)
Supplement: Supplementary file 5 — Correlations between the studied traits of genotypes differentiated in terms of phenology (p < 0.01) (DOCX 14 kb) [file 13353_2023_755_MOESM5_ESM.docx]

The effects of heading time on yield performance and *HvGAMYB* expression in spring barley subjected to drought

Piotr Ogrodowicz*, Anetta Kuczyńska, Paweł Krajewski, Michał Kempa

Institute of Plant Genetics of the Polish Academy of Sciences, Strzeszyńska 34, 60-479 Poznań, Poland

*Corresponding authors:

Tel.: (+48 61) 65 50 224; e-mail: pogr@igr.poznan.pl

| Traits | Tn | PTn | LSm | NSSm | NGSm | WGSm | LSl | NSSl | NGSl | WGSl | GY | TGW | FSm | FSl |
| --- | --- | --- | --- | --- | --- | --- | --- | --- | --- | --- | --- | --- | --- | --- |
| Tn | 1.00 |  |  |  |  |  |  |  |  |  |  |  |  |  |
| PTn | 0.79 | 1.00 |  |  |  |  |  |  |  |  |  |  |  |  |
| LSm |  |  | 1.00 |  |  |  |  |  |  |  |  |  |  |  |
| NSSm |  |  | 0.81 | 1.00 |  |  |  |  |  |  |  |  |  |  |
| NGSm |  |  | 0.68 | 0.71 | 1.00 |  |  |  |  |  |  |  |  |  |
| WGSm | -0.47 |  | 0.74 | 0.69 | 0.92 | 1.00 |  |  |  |  |  |  |  |  |
| LSl |  |  | 0.59 | 0.42 | 0.56 | 0.51 | 1.00 |  |  |  |  |  |  |  |
| NSSl |  |  | 0.49 | 0.60 | 0.55 | 0.46 | 0.86 | 1.00 |  |  |  |  |  |  |
| NGSl |  |  | 0.56 | 0.54 | 0.84 | 0.72 | 0.76 | 0.73 | 1.00 |  |  |  |  |  |
| WGSl | -0.47 |  | 0.61 | 0.49 | 0.81 | 0.84 | 0.74 | 0.63 | 0.91 | 1.00 |  |  |  |  |
| GY |  |  | 0.56 | 0.41 | 0.79 | 0.81 | 0.59 | 0.48 | 0.82 | 0.89 | 1.00 |  |  |  |
| TGW | -0.71 | -0.40 | 0.53 | 0.41 | 0.45 | 0.76 |  |  |  | 0.61 | 0.54 | 1.00 |  |  |
| FSm |  |  |  |  | 0.80 | 0.68 | 0.41 |  | 0.69 | 0.68 | 0.77 |  | 1.00 |  |
| FSl |  |  | 0.39 |  | 0.73 | 0.65 | 0.39 |  | 0.82 | 0.77 | 0.77 |  | 0.78 | 1.00 |

Supplementary File 5. Correlations between the studied traits of genotypes differentiated in terms of phenology (p < 0.01)
