# Supplementary material for: The effects of heading time on yield performance and HvGAMYB expression in spring barley subjected to drought
Source: J Appl Genet. 2023 Mar 10;64(2):289–302. doi: 10.1007/s13353-023-00755-x (PMC10076406; doi:10.1007/s13353-023-00755-x)
Supplement: Supplementary file 7 — Correlations between the studied OJIP parameters of genotypes differentiated in terms of phenology (p value < 0.01) recorded at development point 1 (A), development point 2 (B) (DOCX 24 kb) [file 13353_2023_755_MOESM7_ESM.docx]

The effects of heading time on yield performance and *HvGAMYB* expression in spring barley subjected to drought

Piotr Ogrodowicz*, Anetta Kuczyńska, Paweł Krajewski, Michał Kempa

Institute of Plant Genetics of the Polish Academy of Sciences, Strzeszyńska 34, 60-479 Poznań, Poland

*Corresponding authors:

Tel.: (+48 61) 65 50 224; e-mail: pogr@igr.poznan.pl

A

| Traits | ABS_RC | TRo_RC | ETo_RC | DIo_RC | Fv_Fm | Psi_o | Phi_Eo | Phi_Do | Pi_Abs |
| --- | --- | --- | --- | --- | --- | --- | --- | --- | --- |
| ABS_RC | 1.00 |  |  |  |  |  |  |  |  |
| TRo_RC | 0.99 | 1.00 |  |  |  |  |  |  |  |
| ETo_RC | 0.48 | 0.57 | 1.00 |  |  |  |  |  |  |
| DIo_RC | 0.97 | 0.91 |  | 1.00 |  |  |  |  |  |
| Fv_Fm | -0.83 | -0.73 |  | -0.94 | 1.00 |  |  |  |  |
| Ψ_o | -0.39 |  | 0.56 | -0.49 | 0.58 | 1.00 |  |  |  |
| Φ_Eo | -0.56 | -0.48 | 0.42 | -0.67 | 0.76 | 0.97 | 1.00 |  |  |
| Φ_Do | 0.87 | 0.78 |  | 0.97 | -0.98 | -0.55 | -0.73 | 1.00 |  |
| Pi_Abs | -0.81 | -0.73 |  | -0.89 | 0.90 | 0.83 | 0.94 | -0.90 | 1.00 |

B

| _Traits_ | ABS_RC | TRo_RC | ETo_RC | DIo_RC | Fv_Fm | Psi_o | Phi_Eo | Phi_Do | Pi_Abs |
| --- | --- | --- | --- | --- | --- | --- | --- | --- | --- |
| ABS_RC | 1.00 |  |  |  |  |  |  |  |  |
| TRo_RC | 0.93 | 1.00 |  |  |  |  |  |  |  |
| ETo_RC |  |  | 1.00 |  |  |  |  |  |  |
| DIo_RC | 0.89 | 0.65 |  | 1.00 |  |  |  |  |  |
| Fv_Fm | -0.63 |  | 0.49 | -0.90 | 1.00 |  |  |  |  |
| Ψ_o | -0.50 |  | 0.76 | -0.61 | 0.61 | 1.00 |  |  |  |
| Φ_Eo | -0.59 |  | 0.72 | -0.73 | 0.73 | 0.98 | 1.00 |  |  |
| Φ_Do | 0.67 |  | -0.39 | 0.94 | -0.98 | -0.57 | -0.70 | 1.00 |  |
| Pi_Abs | -0.75 | -0.51 | 0.57 | -0.87 | 0.82 | 0.92 | 0.97 | -0.81 | 1.00 |

Supplementary File 7. Correlations between the studied OJIP parameters of genotypes differentiated in terms of phenology (p value < 0.01) recorded at development point 1 (A), development point 2 (B)
